# FTIR Spectroscopy Analysis of Bound Water in Dried Saliva Samples: Differentiation of Smoking and Non-Smoking Groups and Implications for Oral Cancer Risk

**DOI:** 10.1177/15330338251317304

**Published:** 2025-05-19

**Authors:** Maria Clara Coelho Ferreira, Vitórya Carvalho Pádua de Magalhães, Thayná Melo de Lima Morais, Felipe Peralta, Pedro Arthur Augusto Castro, Denise Maria Zezell, Marcelo Saito Nogueira, Luis Felipe CS Carvalho

**Affiliations:** 1 Departamento de Odontologia, 67891 Universidade de Taubaté, Taubaté, Brazil; 2 Departamento de Odontologia, 355848 UNISOCIESC, Centro Universitário Tupy, Joinville, Brazil; 3 Center for Lasers and Applications, 119500 Instituto de Pesquisas Energeticas e Nucleares, IPEN—CNEN/SP, Sao Paulo, SP, Brazil; 4 261183 Tyndall National Institute, Lee Maltings, Dyke Parade, Cork, Ireland; 5 Department of Physics, University College Cork, College Road, Cork, Ireland; 6 Departamento de Odontologia, Centro Universitário Braz Cubas, Mogi das Cruzes, Brazil

**Keywords:** biomarker, high wavenumber, saliva, smoker, FTIR spectroscopy, optical biopsy, oral cancer, oral pathology, Fourier transform infrared spectroscopy, vibrational spectroscopy

## Abstract

**Background:** According to the WHO, oral cancer is the thirteenth most common cancer worldwide, with tobacco use being one of the primary causes of oral cancer. This study aimed to characterize and differentiate the saliva and bound water using FTIR spectroscopy in smoking and non-smoking individuals. **Materials and Methods:** This prospective observational study analyzed dried saliva samples from control, smoking, and occasional smoking groups using an attenuated total reflectance Fourier Transform Infrared (ATR-FTIR) spectrometer. The high wavenumber spectral region of 2800–3600 cm-¹ was selected for analysis. **Results:** The results indicate that standard variance normalization (SNV) reduced intragroup variability and highlighted differences in smokers’ spectra within the 3250–3500 cm-¹ region, associated with the absorption of water bound to saliva molecules. Cubic SVM models using SNV spectra demonstrated higher classification accuracy between groups, achieving 15.6% greater sensitivity and 1.3% lower specificity compared to models based on the second-order derivative. RUSBoosted Trees addressed data imbalances, enhancing both sensitivity and specificity. The study suggests that spectral changes may reflect salivary biochemistry linked to smoking and potentially to oral cancer risk. **Conclusions:** We conclude that differentiation between normal individuals and smokers can be achieved using high wavenumber FTIR spectral analysis. Additionally, we demonstrate the relationship between bound water molecules and salivary biomolecules in control, smoking, and occasional smoking groups. This technique has potential applications in elucidating OH vibrations within biological systems.

## Introduction

According to the WHO, oral cancer is the thirteenth most common cancer worldwide, with a global incidence of 377,713 new cases. Tobacco use is among the main causes of oral cancer. In Brazil, where the study was conducted, the estimated numbers of new oral cancer cases are 10.30 new cases per 100,000 men and 3.83 per 100,000 women (INCA, 2023). Considering this data, we conducted this pilot study with the aim of characterizing the saliva of smoking and non-smoking patients through FTIR so that these data may soon be used as identifiers of potential oral cancer.

FTIR spectroscopy consists of illuminating samples with polychromatic light and measuring their infrared light absorption. Fourier transform is used to convert the raw data collected into absorption spectra.^2^ FTIR spectroscopy has been researched as a potential alternative to laboratory testing due to its high sensitivity and specificity for analyzing body fluids such as blood, saliva and urine.^3,4^ In this context, FTIR spectroscopy has the potential to be used for detection, diagnosis, and monitoring of diseases, analyzing the chemical composition of cells and tissues.^2^ FTIR spectroscopy advantages include ease of instrumentation, minimal sample preparation, small sample volumes and real-time in vitro diagnostics. As a method of vibrational optical spectroscopy,^5^ FTIR spectroscopy can non-invasively characterize biomolecules^6^ and is commonly used to differentiate healthy and pathological tissues.^7,8^

Another important detail is that the evaluation of the region of high wavenumbers in vibrational spectroscopy is very important for the discrimination of healthy and pathological samples, and sometimes with more important and precise information when we evaluate the fingerprint region (most used). We can observe some works previously published by our research group, even involving research using Raman spectroscopy, the information and spectral details related to the biochemical characteristics are extremely similar.^9,10^

An indirect biochemical assessment of samples can evaluate the molecular binding of water by using high-wavenumber FTIR spectroscopy. Since water binds to specific substances in biofluids, its binding can be a potential biomarker for understand the pathophysiology of many diseases.^9^ In this study, we evaluated the change in the molecular binding of water molecules resulting from pathological processes induced by smoking. We assessed water binding in smoker and non-smoker patients based on spectral differences in the OH vibrations in the high wavenumber region between 3000–3600 cm^−1^. We first evaluated the classification performance of sample machine learning models (classifiers) to differentiate FTIR spectroscopy. Diagnostic biomarker in the correlated regions were also presented.

## Materials and Methods

### Clinical Protocol and Research Ethics

This was a prospective observational study, which followed the guidelines of the Strengthening Reporting of Observational Studies in Epidemiology (STROBE) statement.^11^ The spit/expectoration method was used to collect the sample. Saliva samples from non-smokers and smokers were collected in a sterile universal collector, homogenized, and stored at −20 °C until analysis. All procedures performed in this study were approved by the Research Ethics Committee of the University of Taubaté under protocol number 19436919.7.0000.5501. The sample consisted of adults who agreed to participate in the research. As exclusion criteria, patients with systemic diseases, minors, and those who did not agree to participate in the research were excluded. All patients included in the study were invited to participate and have consented to signing an informed consent form.

### Data Collection

FTIR measurements were conducted using an ATR FTIR Spectrometer (Nicolet 6700, ThermoFisher Scientific) to analyze dried saliva samples. By completely drying every saliva sample, we ensured that the remaining water was bound to organic saliva constituents such as proteins. In addition, lipids and carbohydrates remaining on dried samples are not affected by water absorption which contaminates FTIR signals. The drying procedure consisted of placing 1 μl of each saliva sample on the crystal with no additives and waiting for complete drying for times between 2–7 min (average time of 5 min). Contamination was avoided by cleaning the crystal with 92.8% alcohol by using absorbent paper. One sample and three spectra per study participant, a total of 32 background scans were performed before each spectrum, and for each spectrum, 32 scans were conducted with a resolution of 4 cm^−1^.

### Data Analysis

The high-wavenumber spectral region from 2800–3600 cm^−1^ was selected for FTIR spectral analysis. To evaluate the existence of statistically significant differences between saliva samples of control (n = 11), smoker (n = 9), and occasional smoker (n = 8) groups, three-way ANOVA and post hoc tests were used to evaluate the area under the curve of FTIR absorption bands for the wavenumber range of specific bands. Metrics of CH, OH, and NH vibrations of water and proteins were considered coming from the 3050–3600 cm^−1^ band, whereas CH2 and CH3 vibrations of lipids and proteins were assumed to originate from the 2800–3050 cm^−1^ spectral region.

We evaluated models for (1) classification of each of the study groups separately (ie, control vs occasional smoker vs smoker groups), (2) classification of the control versus smoker groups, and (3) classification of a combined control and occasional smoker groups versus smoker group. For all cases, we presented the result of the most accurate classification model while still prioritizing a balanced sensitivity-to-specificity ratio for clinical purposes. To mitigate the influence of the imbalanced dataset in our classification of controls & occasional smokers (n = 19) versus smokers (n = 9), models performing this classification had their performance assessed by metrics achieved by random under-sampling boosting decision trees in a RUSBoosted Trees Ensemble model (in addition to showing the results for the most accurate model).

Classification performance metrics were calculated by building classification models based on multiple machines learning algorithms and evaluating their performance by using 5-fold cross-validation. Machine learning algorithms tested included fine, medium and coarse (decision) tree algorithms, linear and quadratic discriminant analysis (LDA and QDA, respectively), linear, quadratic, cubic, fine Gaussian, medium Gaussian and coarse Gaussian support vector machines (SVM), fine, medium, coarse, cosine, cubic and weighted K-nearest neighbors (KNN), and others.

## Results

### Effect of Data Preprocessing Methods in High-Wavenumber spectra

**Figure 1.**
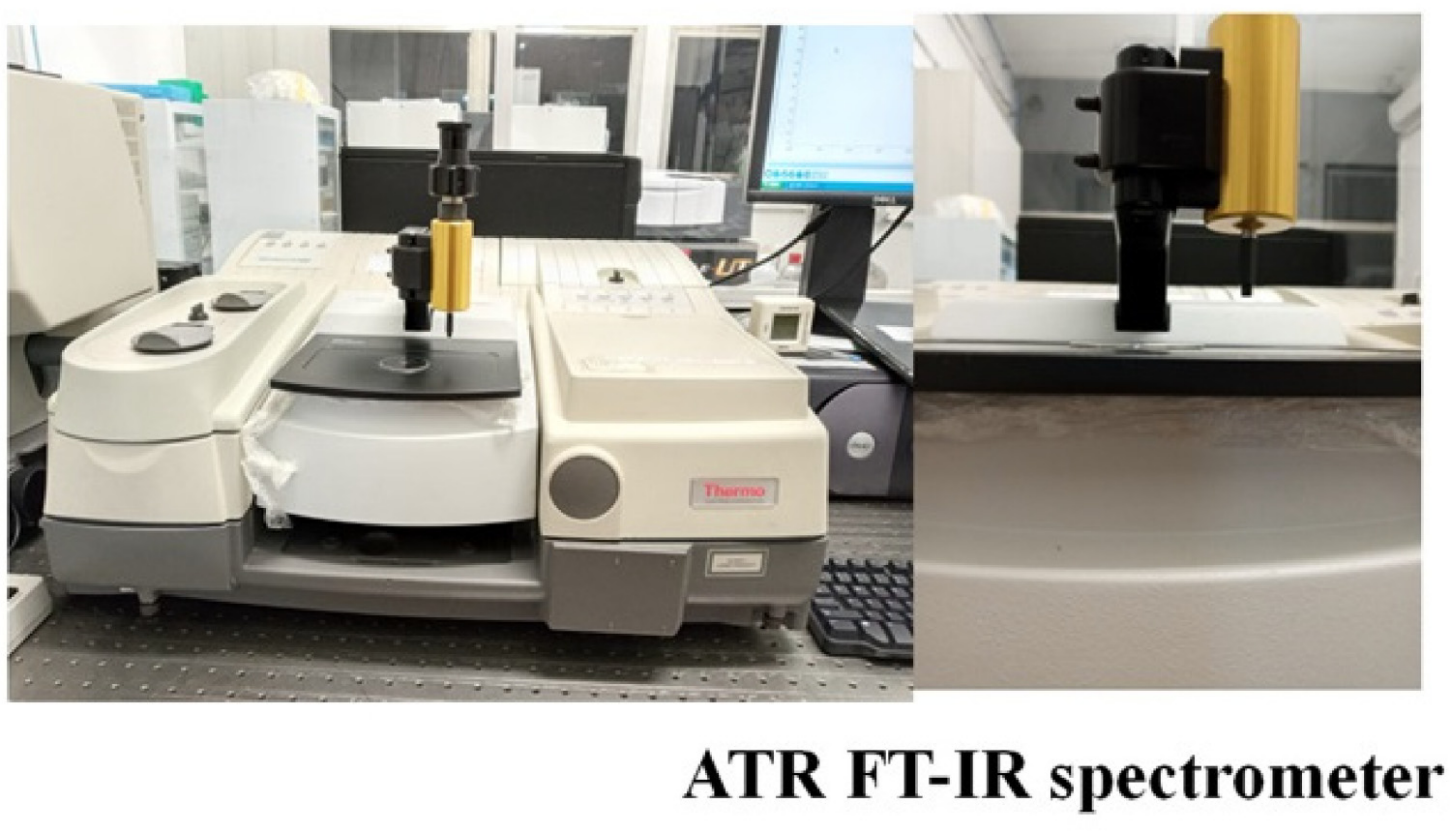
Shows the equipament and the crystal where the sample is dried.

Figure 1 shows the equipament and the crystal where the sample is dried.

**Figure 2.**
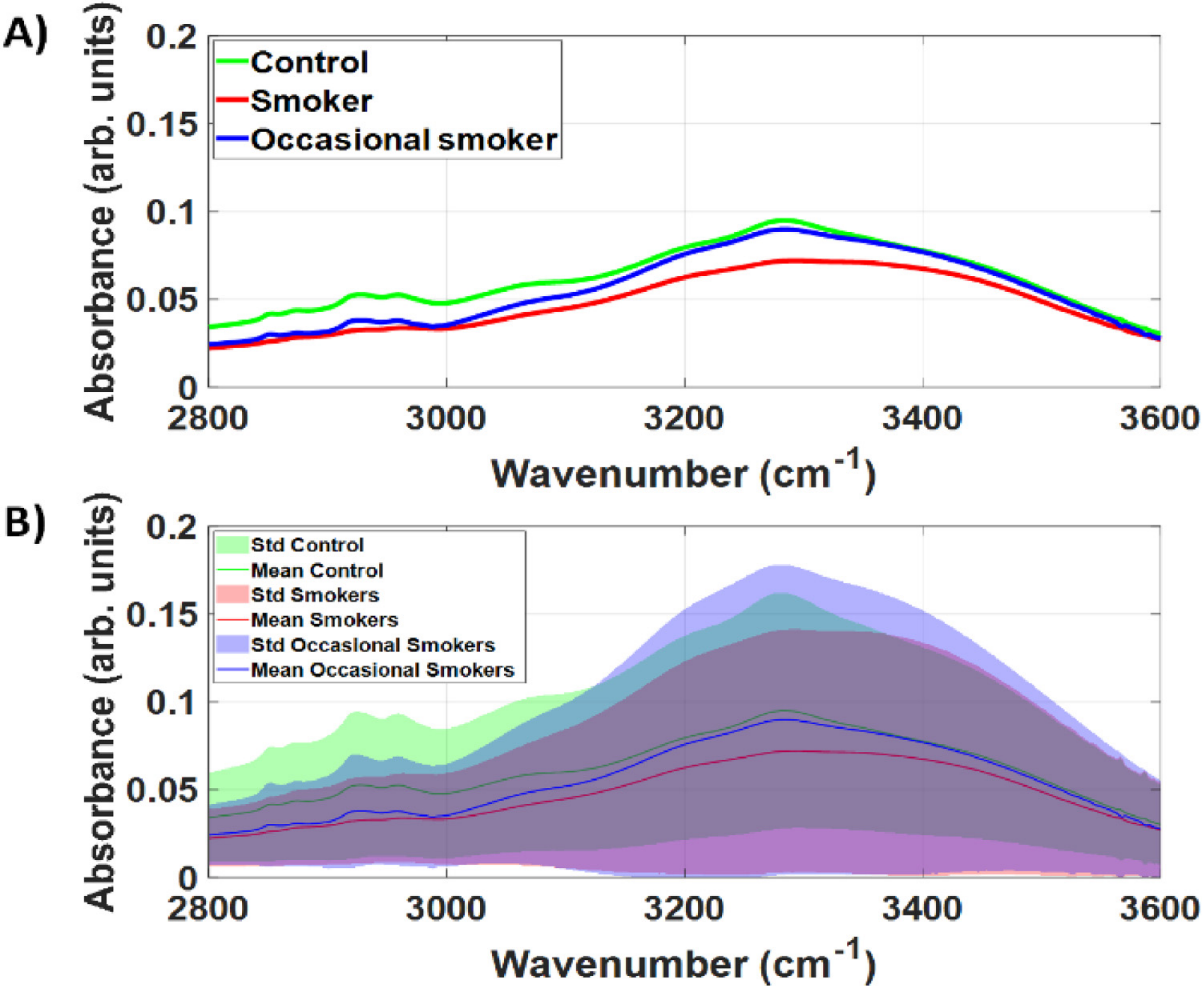
A) and B) Mean and standard deviation of high-wavenumber region of the raw FTIR spectra collected in this study.

Figure 2 shows that the spectral mean and standard deviation of each study group overlap considerably. The most prominent and broad peak is the OH vibration of bound water, which is bound to molecules such as proteins of the dried saliva samples. Other characteristic saliva bands are present at 2850 cm^−1^ (CH3 symmetric stretching; vsCH3), 2875 cm^−1^ (vsCH3), 2930 cm^−1^ (CH2 asymmetric stretching; vasCH2), 2959 cm^−1^ (CH3 asymmetric stretching; vasCH3) and correspond to lipids, fatty acids and DNA. Less prominent bands appear as a shoulder of the broad OH spectral band. These bands are centered at 3070 cm^−1^ (vsCH3), 3207 cm^−1^ (NH symmetric stretching; vsNH), 3288 cm^−1^ (NH stretching of amide A and OH stretching of carbohydrates), and 3410 cm^−1^ (OH asymmetric stretching).^12,13^

**Figure 3.**
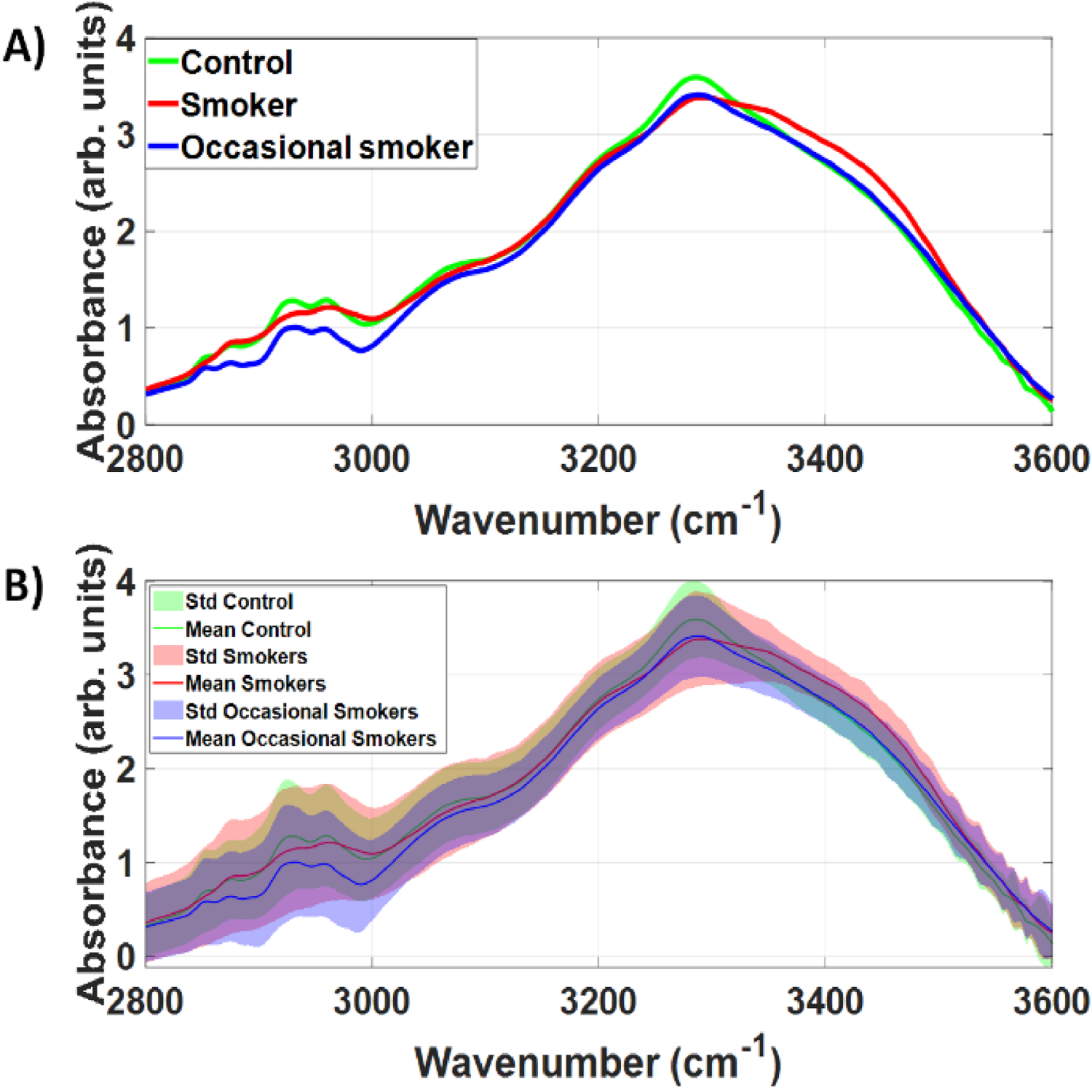
A) Mean and B) Mean and standard deviation of the Standard Normal Variate (SNV) of the raw FTIR spectra collected in this study. For all study groups, the standard deviation of SNV is significantly smaller than the standard deviation of the raw FTIR spectra in the high-wavenumber region.

Figure 3 indicates that SNV can reduce the standard deviation between saliva spectra of each study group. While FTIR spectra varies less intragroup, we still observed an overlap between spectra of each group. However, the overlap between control and smoker groups was smaller at wavenumbers between 3250–3500 cm^−1^. A smaller overlap in this including cancerous changes) could be detected by alterations in water absorption induced by binding with smoking-related saliva molecules in such wavenumber range.

**Figure 4.**
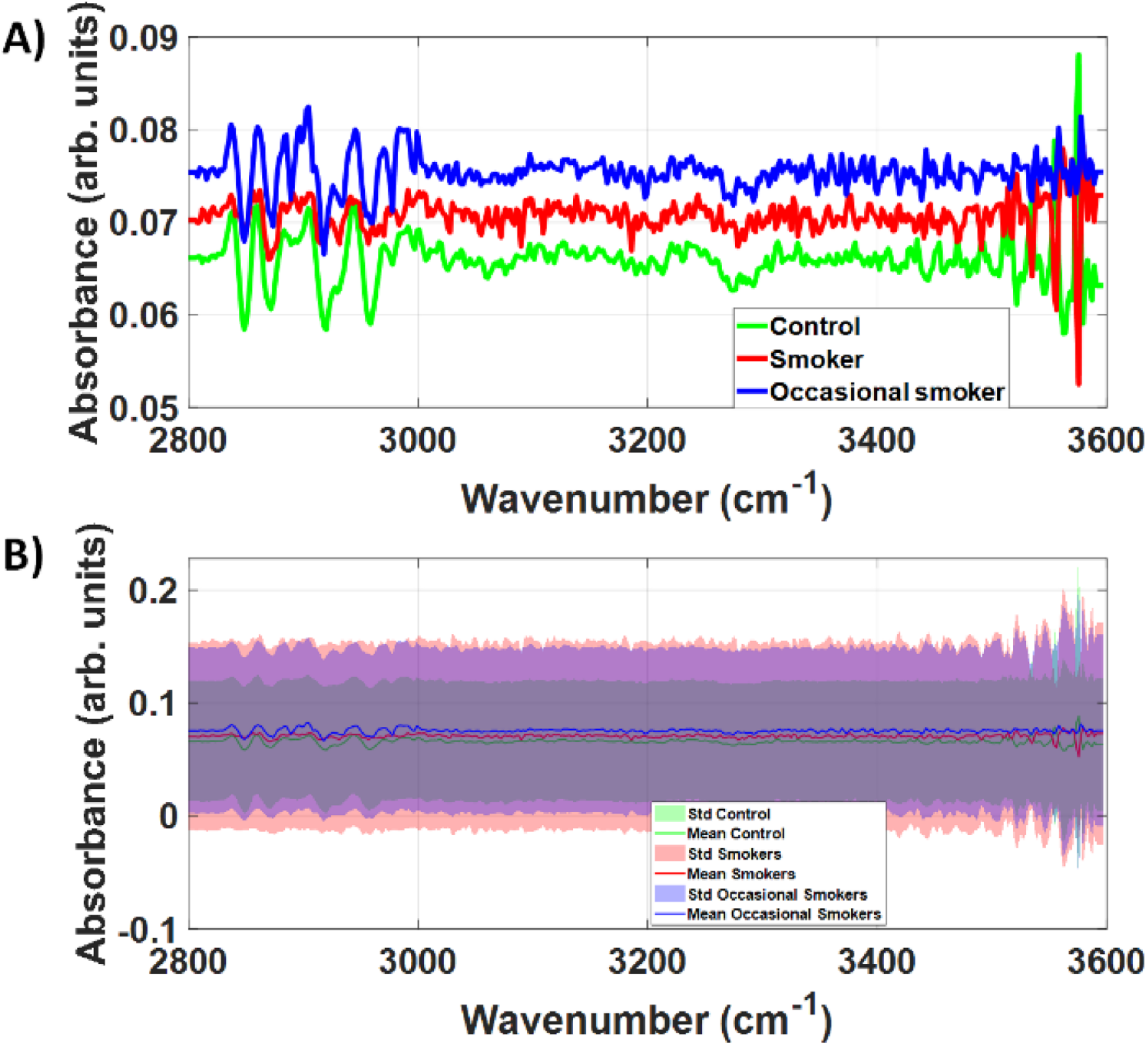
A) Mean and B) Mean and standard deviation of the second derivative spectra of SNV of the raw FTIR spectra. For all study groups, the standard deviation of the second derivative spectra was significantly larger than the standard deviation of the raw FTIR spectra in the high-wavenumber region.

Figure 4 shows that second derivative of the SNV spectra of all study groups had a larger standard deviation compared to the raw FTIR spectra and SNV spectra. Our results indicate that high frequency spectral components would not significantly contribute to increase the accuracy of smoking detection.

**Figure 5.**
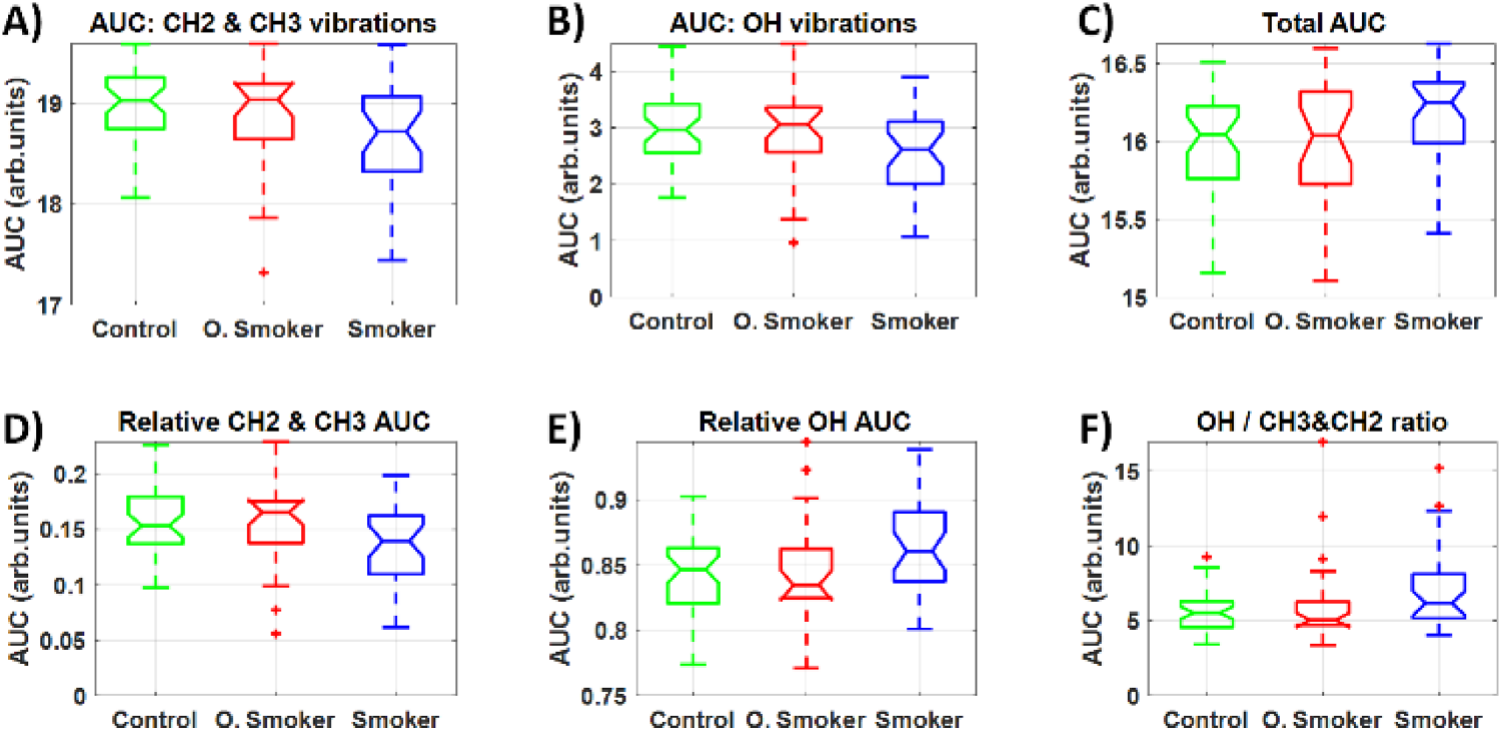
A) Area under the curve (AUC) of CH2 and CH3 vibrational spectral bands, B) OH vibrational spectral bands, C) both OH, CH2 and CH3 vibrational spectral bands (ie, total AUC between 2800–3600 cm-1), D) AUC of CH2 and CH3 vibrational spectral bands normalized by the total AUC, E) AUC of OH vibrational spectral bands normalized by the total AUC, and the ratio between metrics of B) and A). All parameters were calculated by using vector normalized spectra. No significant statistical difference was found between study groups.

Figure 5 indicates that no significant statistical difference could be observed between absolute values of area under the curve (AUC) for the OH, CH2 and CH3 bands, their ratio and their relative value compared to the total AUC for each sample.

### Patient Sample Classification

Tables 1 and 2 show that the cubic SVM model was most accurate to classify study groups based on SNV spectra, whereas the linear SVM was most accurate model for classification using the second derivative spectra. When building classification models for control, occasional smoker and smoker groups, the cubic SVM model based on SNV spectra was more accurate to classify between control and smoker groups. Similarly, the cubic SVM model based on second derivative spectra was more accurate to classify between occasional smoker and smoker groups. Therefore, the high frequencies of SNV spectra featured in its second derivative may be associated with the biochemistry of smoking frequency and dependence.

**Table 1.** Confusion Matrix of the Cubic SVM Model Using SNV spectra.

		Predicted		
		Control (n = 11)	Occasional Smokers (n = 8)	Smokers (n = 9)
**True class**	**Control**	66.1%	25.9%	7.8%
	**Occasional Smokers**	15.9%	53.3%	18.5%
	**Smokers**	18.0%	20.8%	73.7%

The confusion matrix shows the percentage of correctly classified observations for control, occasional smoker and smoker groups.

**Table 2.** Confusion Matrix of the Linear SVM Model Using the Second Derivative of SNV Spectra.

		Predicted		
		Control (n = 11)	Occasional Smokers (n = 8)	Smokers (n = 9)
**True class**	**Control**	46.5%	19.9%	28.8%
	**Occasional Smokers**	28.6%	72.8%	6.8%
	**Smokers**	24.9%	7.3%	64.4%

The confusion matrix shows the percentage of correctly classified observations for control, occasional smoker and smoker groups.

Since SNV spectra were more accurate to classify control and smoker groups, we evaluated the increase in sensitivity and specificity obtained by considering both low and high frequencies of the SNV spectra. This evaluation includes a comparison between results of the most accurate classification models based on SNV spectra and second derivative spectra for only control and smoker groups. Tables 3 and 4 indicate that the cubic SVM model based on SNV spectra achieved 15.6% more sensitivity and 1.3% less specificity compared to the medium Gaussian SVM model based on second derivative spectra.

**Table 3.** Confusion matrix of the cubic SVM model using SNV spectra. The confusion matrix shows the percentage of correctly classified observations for control and smoker groups

		Predicted	
		Control (n = 11)	Smokers (n = 9)
**True class**	**Control**	81.4%	12.0%
	**Smokers**	18.6%	88.0%

**Table 4.** Confusion matrix of the medium Gaussian SVM model using the second derivative of SNV spectra.

		Predicted	
		Control (n = 11)	Smokers (n = 9)
**True class**	**Control**	**82.7%**	**27**.**6%**
	**Smokers**	**17.3%**	**72**.**4%**

The confusion matrix shows the percentage of correctly classified observations for control and smoker groups.

In addition, we evaluated the classification of controls & occasional smokers (n = 19) versus smokers (n = 9) to assess whether major differences in saliva samples appeared only after high smoking frequency. By combining control and occasional smoker data, we show below results with an increased sample size while having an imbalanced dataset for classification versus the smoker group. For comparison with the control versus smokers’ model, Tables 5 and 6 indicate results of a quadratic SVM and a cubic SVM model for classification using the SNV spectra and their second derivative, respectively. Due to the imbalanced dataset and the low-frequency features lost in the second derivative spectra, we observed that SVM models including cubic SVM do not achieve balanced sensitivity-to-specificity ratios (please see the Supplemental material for more information on other SVM models). In particular, a model with sensitivity-to-specificity ratios far from 1 (unity) such as the model obtained for the second derivative spectra (16.5% sensitivity and 94.3% specificity) are not useful clinically.

**Table 5.** Confusion matrix of the quadratic SVM model using SNV spectra.

		**Predicted**	
		**Control & Occasional smokers (n = 19)**	**Smokers (n = 9)**
**True class**	**Control**	88.2%	32.9%
	**Smokers**	11.8%	67.1%

The confusion matrix shows the percentage of correctly classified observations for control and occasional smokers group versus smoker group.

**Table 6.** Confusion matrix of the cubic SVM model using the second derivative of SNV spectra.

		Predicted	
		Control & Occasional smokers (n = 19)	Smokers (n = 9)
**True class**	**Control**	94.3%	83.5%
	**Smokers**	5.7%	16.5%

The confusion matrix shows the percentage of correctly classified observations for control and occasional smokers group versus smoker group.

Finally, Table 7 show results RUSBoosted Trees Ensemble model to mitigate the influence of the imbalanced dataset in our classification. For such model, we observed 0.7% higher specificity and 3.7% sensitivity for the model based on SNV spectra compared to the model based on second derivative spectra.

**Table 7.** Confusion matrix of the RUSBoosted Trees Ensemble model using the second derivative of SNV spectra.

		Predicted	
		Control & Occasional smokers (n = 19)	Smokers (n = 9)
**True class**	**Control**	75.4%	36.1%
	**Smokers**	24.6%	63.9%

The confusion matrix shows the percentage of correctly classified observations for control and occasional smokers’ group versus smoker group.

Hence, by using multiple classification models on metrics of FTIR saliva spectra, we could potentially not only assess individual smoking status but also unveil the bound water biochemistry associated with smoking. Similarly, this assessment may provide information on the risk of oral cancer since the smoking status is closely related to oral squamous cell carcinoma.

## Discussion

FTIR spectroscopy was used to analyze water confinement in a high wavenumber region and determine its intrinsic molecular changes, since analysis of vibrational modes compared to literature results showed that the inflammatory process leads to changes in the collagen-related high-wavenumber spectral region and confined water^14^ as well as the performance of sample differentiation using various types of classifiers. Oral lesions are currently diagnosed by the histopathological analysis of the biopsy, which is an invasive procedure and does not present immediate results.^15^ Thus, FTIR spectroscopy has significant advantages as it is non-invasive and provides rapid diagnosis, enabling early diagnosis. Considering that early diagnosis of oral cancer can significantly improve patient survival rate and reduce medical costs.^16^

Accordingly infrared absorption spectroscopies have, in general, attracted a lot of attention over the past decades for biomedical applications, and particularly, more recently for analysis of biofluids, including, saliva, bile, breast milk, pancreatic juice, tears, blood, urine.^17^ The fingerprint spectral region (400-1800cm−1) has been shown to be very promising for optical biopsy purposes. However, limitations for discrimination of dysplastic and inflammatory processes based on the fingerprint region have been demonstrated. In addition, the high wavenumber region (2800-3600 cm−1) provides more specific information based on N-H, O-H and C-H vibrations and can be used to identify the subtle changes which could be important for discrimination of samples.^10,18^

As mentioned, FTIR measurements were performed using an ATR FTIR spectrometer to measure dry saliva samples, because drying each saliva sample ensures that the remaining water is bound to organic constituents of saliva and lipids and carbohydrates are not affected by the absorption of water that contaminates the signals. In this context, the present work shows the relationship of the spectral region of the high wave numbers with the vibrations of OH, which show how confined water can be altered in different sample groups, in the study of smokers, sporadic smokers and non-smokers.

Our results suggest that confined water spectral changes can be observed mainly in the high wavenumber regions in the 2800–3600 cm^−1^ bands, so it was used for FTIR spectral analysis. Thus, the existence of statistically significant differences between saliva samples from the control, smoker and occasional smoker groups was evaluated, these alterations may be associated with oral cancer, as smoking is a risk factor.^1^ In general, FTIR spectroscopy was effective in differentiating these groups. In this context, metrics of CH, OH and NH vibrations of water and proteins were considered to originate from the 3050–3600 cm^−1^ band, while the CH2 and CH3 vibrations of lipids and proteins were considered to originate from the spectral region of 2800–3050 cm^−1^.

The most prominent and broad peak was observed to be the OH vibration of bound water, which is confined to molecules such as proteins from the dried saliva samples. Other characteristic salivary bands are present at 2850 cm^−1^ (CH3 symmetrical elongation; vsCH3), 2875 cm^−1^ (vsCH3), 2930 cm^−1^ (CH2 asymmetrical elongation; vasCH2), 2959 cm^−1^ (CH3 asymmetrical elongation; vasCH3) and correspond to lipids, fatty acids, and DNA. In addition, less prominent bands appear as a shoulder of the broad OH spectral band. These bands are centered at 3070 cm^−1^ (vsCH3), 3207 cm^−1^ (symmetric NH stretch; vsNH), 3288 cm^−1^ (amide A NH stretch and carbohydrate OH stretch) and 3410 cm^−1^ (asymmetric OH stretch). Next steps include increasing the number of volunteers involved in the study to incorporate more data on biological variability into classification models and for future exploratory analysis.

Optical spectroscopies encompass a set of methods that measure the values of light/matter interaction phenomena, using light to study molecular processes. In this context, vibrational spectroscopies (Raman scattering and infrared absorption) are optical spectroscopy techniques based on transitions between vibrations within the same electronic state.^19^ Consequently, it's possible to deduce information about the nature and structure of a molecule, whether in free or bound form, as well as its interaction with the environment. One significant application is in the identification and characterization of cancerous and non-cancerous tissues, particularly in breast tissue.^20^

Water can generate small active clusters and macroscopic assemblies that can transmit information at different scales.^20^ The most prominent and broad peak is the water-bound OH vibration, which is linked to molecules such as proteins in samples of dried saliva. Other characteristic saliva bands are present at 2850 cm^−1^, 2875 cm^−1^, 2930 cm^−1^, 2959 cm^−1^, corresponding to lipids, fatty acids, and DNA. In this sense, each water molecule not only forms up to four directed hydrogen bonds with neighboring water molecules but can also establish dipole-dipole and dipole-induced interactions with other molecules. Such water clusters give liquid water a heterogeneous character that can change with different physical and environmental conditions. Biomolecule atoms can replace any or all of the bonds around each water molecule, affecting the structuring of adjacent water molecules and biomolecular groups as well as secondary bonded molecules and distant parts of biomolecules.^21,22^

Thus, in the high wavenumber spectral region of 2800–3600 cm^−1^, through FTIR analysis, it is possible to describe confined water. This is of great relevance and applicability in the medical field, as increased cellular hydration can promote both tumor growth and metastasis. In this sense, a progressive increase in cellular hydration, induced by successive genetic changes or epigenetic alterations, is the basic mechanism of multistage carcinogenesis^23,24^; moreover, the degree of malignancy increases with the degree of cellular hydration.^23^ Considering the above, these hypotheses imply that increased cellular hydration is a common factor that promotes both tumor growth and metastasis, and that the metastatic potential increases with the degree of cellular hydration.^23^ Increased cellular hydration is also proposed as an alternative or additional explanation for the carcinogenic effect of inflammatory agents and hormones.^21^

The advantage of “optical biopsy” lies in providing direct biochemical information (vibrational fingerprint) in real-time, at the point of care, without subjective interpretations. FTIR spectroscopy monitors biological tissue structures without external agents, contrasting with histological evaluation.^19^ This safe, rapid, and accurate diagnostic technique benefits clinician, allowing non-invasive, on-the-spot disease diagnosis, reducing patient anxiety and emotional strain.

In a study focusing on the diagnosis of breast tissue cancer using Raman spectroscopy, pronounced differences were observed in the biochemical composition and distribution of carotenoids (1158 and 1518 cm^−1^), lipids (2800-3000 cm^−1^), and water represented by the OH stretching band at 3311 cm^−1^. It is crucial to emphasize that comparing Raman spectra with infrared spectra provides complementary information about characteristic vibrations in normal and cancerous tissues.^20^

With the above in mind, once we have determined which vibrational modes are similar between the FTIR spectra of the saliva of smokers and cancer patients, following the presence of these vibrational modes in a larger population and over a longer period of time (eg, in 2 or 5 years follow-up studies) will allow us to investigate which modes and corresponding biochemical changes (biomarkers) are associated with the development of oral cancer from smoking as its main risk factor. At this stage, FTIR spectroscopy can be used as a point-of-care (POC) diagnostic tool to identify oral cancer biomarkers once the FTIR technology is sufficiently mature to produce compact and cost-effective instruments. These instrument production aspects combined with the small sample volume required and the real-time sample analysis provided by FTIR spectroscopy potentially allow for faster clinical translation and commercialization as key steps towards implementing POC technologies for screening tests of high yield.

In summary, it is worth mentioning that a requirement for clinical translation of the FTIR and training of clinicians who can use it daily in the clinic is necessary. Besides that, software development professionals should work on creating user-friendly software interfaces for healthcare professionals. It is also interesting to understand and stimulate the interest of the industries that produce this equipment to work directly within hospitals and clinics, and alongside health professionals, so that they would understand closely what could be improved.^18^

Therefore, the early engagement of multiple clinical research centers and the implementation of robust machine learning methods to provide individualized oral cancer risk scores, even with preliminary datasets, would greatly enhance the involvement of clinicians in multicenter studies. Such studies could potentially culminate in the development of technologies applicable to national and international cancer screening programs. For instance, the use of ATR-FTIR spectroscopy combined with chemometrics, as demonstrated in lung cancer detection studies within the NHS England screening program, highlights the feasibility of integrating real-world, noninvasive approaches into clinical workflows. Similarly, saliva-based FTIR spectroscopy could play a pivotal role in detecting early-stage oral cancers, aligning with methodologies already employed in lung cancer screening initiatives.^25^ The benefits to patients would be substantial, ranging from early cancer detection to significantly improved prognoses. Early-stage diagnosis often leads to better treatment outcomes, as demonstrated in lung cancer studies, and enables targeted interventions before symptoms appear.^25^ Clinicians also benefit by effectively managing a larger number of patients, as FTIR spectroscopy offers a noninvasive and non-destructive alternative to conventional biopsies, which require histopathological analysis and are inherently invasive.

In the long term, initiating cancer treatments at an early stage has the potential to transform patient outcomes, particularly by addressing barriers related to socioeconomic disparities.^26–28^ Integrating noninvasive diagnostic tools, such as FTIR spectroscopy, into screening programs for cancers like oral and lung cancers can ensure equitable access to early detection, ultimately improving survival rates across diverse populations.

We emphasize that a more robust evaluation of the potential of FTIR for patient stratification requires increasing the number of patients of our study, especially achieving more solid results when including more participants. The technique's limitations are primarily related to the quantification of molecules, for which alternative analytical methods may be applied, and the cost of the equipment. Additionally, we emphasize the need for further studies utilizing this technology in clinical settings with larger sample sizes to strengthen the technique and enhance the medical team's expertise, ultimately benefiting patients.

## Conclusions

We concluded that differentiating normal and smoking individuals may be performed by using high-wavenumber FTIR spectral analysis. In addition to this observation, we can show the relationship of water molecules bound to saliva biomolecules for control, smoker, and occasional smoker groups. It was possible to conclude that the technique could be used to elucidate OH vibrations inside biological systems.

## Supplemental Material

sj-docx-1-tct-10.1177_15330338251317304 - Supplemental material for FTIR Spectroscopy Analysis of Bound Water in Dried Saliva Samples: Differentiation of Smoking and Non-Smoking Groups and Implications for Oral Cancer Risk Supplemental material, sj-docx-1-tct-10.1177_15330338251317304 for FTIR Spectroscopy Analysis of Bound Water in Dried Saliva Samples: Differentiation of Smoking and Non-Smoking Groups and Implications for Oral Cancer Risk by Maria Clara Coelho Ferreira, Vitórya Carvalho Pádua de Magalhães, Thayná Melo de Lima Morais, Felipe Peralta, Pedro Arthur Augusto Castro, Denise Maria Zezell, Marcelo Saito Nogueira and Luis Felipe CS Carvalho in Technology in Cancer Research & Treatment

## Abbreviations

WHO: World Health Organization
FTIR: Fourier transform infrared spectroscopy.
