# Supplementary material for: FTIR Spectroscopy Analysis of Bound Water in Dried Saliva Samples: Differentiation of Smoking and Non-Smoking Groups and Implications for Oral Cancer Risk
Source: Technol Cancer Res Treat. 2025 May 19;24:15330338251317304. doi: 10.1177/15330338251317304 (PMC12089716; doi:10.1177/15330338251317304)
Supplement: sj-docx-1-tct-10.1177_15330338251317304 - Supplemental material for FTIR Spectroscopy Analysis of Bound Water in Dried Saliva Samples: Differentiation of Smoking and Non-Smoking Groups and Implications for Oral Cancer Risk [file sj-docx-1-tct-10.1177_15330338251317304.docx]

**Supplementary material**

**Table 1S:** Classification performance metrics for all models using SNV spectra to classify control versus smoker groups.

|  | **Classification performance metric** | | | | |
| --- | --- | --- | --- | --- | --- |
| **Classification model** | **Specificity** | **Sensitivity** | **Accuracy** | **AUROC_Control & Occasional smokers** | **AUROCSmokers** |
| **Fine Tree** | 0.64634 ± 0.041497 | 0.69394 ± 0.083366 | 0.66757 ± 0.04004 | 0.68816 ± 0.043135 | 0.68816 ± 0.043135 |
| **Medium Tree** | 0.63902 ± 0.052251 | 0.68788 ± 0.10409 | 0.66081 ± 0.041801 | 0.67838 ± 0.057823 | 0.67853 ± 0.058121 |
| **Coarse Tree** | 0.68659 ± 0.058298 | 0.63788 ± 0.081356 | 0.66486 ± 0.049912 | 0.67199 ± 0.054896 | 0.67199 ± 0.054896 |
| **Linear Discriminant Analysis** | 0.84268 ± 0.053363 | 0.67879 ± 0.034621 | 0.76959 ± 0.032948 | 0.76074 ± 0.031374 | 0.76074 ± 0.031374 |
| **Quadratic Discriminant Analysis** | 0.85122 ± 0.041798 | 0.67273 ± 0.060286 | 0.77162 ± 0.030026 | 0.76197 ± 0.031363 | 0.76197 ± 0.031363 |
| **Linear SVM** | 0.75 ± 0.040253 | 0.74545 ± 0.054208 | 0.74797 ± 0.032596 | 0.82742 ± 0.024251 | 0.82742 ± 0.024251 |
| **Quadratic SVM** | 0.85 ± 0.03289 | 0.69394 ± 0.041596 | 0.78041 ± 0.026624 | 0.85902 ± 0.019083 | 0.85902 ± 0.019083 |
| **Cubic SVM** | 0.91707 ± 0.039881 | 0.73788 ± 0.060825 | 0.83716 ± 0.033812 | 0.88777 ± 0.027225 | 0.88777 ± 0.027225 |
| **Fine Gaussian SVM** | 0.93049 ± 0.027725 | 0.23333 ± 0.061477 | 0.61959 ± 0.026768 | 0.74268 ± 0.038602 | 0.74268 ± 0.038602 |
| **Medium Gaussian SVM** | 0.80244 ± 0.041042 | 0.63485 ± 0.061771 | 0.7277 ± 0.042116 | 0.81789 ± 0.030999 | 0.81789 ± 0.030999 |
| **Coarse Gaussian SVM** | 0.94512 ± 0.022206 | 0.26061 ± 0.050516 | 0.63986 ± 0.024519 | 0.73271 ± 0.020493 | 0.73271 ± 0.020493 |
| **Fine KNN** | 0.81585 ± 0.053363 | 0.58636 ± 0.063164 | 0.71351 ± 0.040327 | 0.70111 ± 0.040835 | 0.70111 ± 0.040835 |
| **Medium KNN** | 0.80976 ± 0.041573 | 0.45455 ± 0.042855 | 0.65135 ± 0.028616 | 0.70275 ± 0.027191 | 0.70275 ± 0.027191 |
| **Coarse KNN** | 1 ± 0 | 0 ± 0 | 0.55405 ± 1.1391e-16 | 0.4745 ± 0 | 0.47228 ± 1.1391e-16 |
| **Cosine KNN** | 0.71463 ± 0.040427 | 0.67424 ± 0.063621 | 0.69662 ± 0.040058 | 0.74821 ± 0.031597 | 0.74821 ± 0.031597 |
| **Cubic KNN** | 0.80488 ± 0.061296 | 0.4303 ± 0.06417 | 0.63784 ± 0.050487 | 0.67228 ± 0.047802 | 0.67228 ± 0.047802 |
| **Weighted KNN** | 0.85122 ± 0.039487 | 0.5197 ± 0.052279 | 0.70338 ± 0.032655 | 0.76016 ± 0.03202 | 0.76016 ± 0.03202 |
| **Boosted Trees Emsemble** | 1 ± 0 | 0 ± 0 | 0.55405 ± 1.1391e-16 | 1 ± 0 | 0 ± 0 |
| **Bagged Trees Emsemble** | 0.79146 ± 0.055661 | 0.65455 ± 0.051464 | 0.73041 ± 0.041006 | 0.78062 ± 0.032457 | 0.78062 ± 0.032457 |
| **RUSBoosted Trees Ensemble** | 0.69268 ± 0.083521 | 0.57576 ± 0.10358 | 0.64054 ± 0.034634 | 0.64618 ± 0.032369 | 0.64427 ± 0.05517 |
| **Subspace Discriminant Ensemble** | 0.74756 ± 0.034742 | 0.75152 ± 0.051651 | 0.74932 ± 0.025743 | 0.84257 ± 0.017044 | 0.84257 ± 0.017044 |
| **Subspace KNN Ensemble** | 0.79512 ± 0.047082 | 0.53485 ± 0.053195 | 0.67905 ± 0.032181 | 0.77012 ± 0.026463 | 0.77012 ± 0.026463 |

**Table 2S:** Classification performance metrics for all models using the second derivative of SNV spectra to classify control versus smoker groups.

|  | **Classification performance metric** | | | | |
| --- | --- | --- | --- | --- | --- |
| **Classification model** | **Specificity** | **Sensitivity** | **Accuracy** | **AUROC_Control & Occasional smokers** | **AUROCSmokers** |
| **Fine Tree** | 0.68415 ± 0.071865 | 0.54242 ± 0.1063 | 0.62095 ± 0.062541 | 0.62045 ± 0.064244 | 0.62045 ± 0.064244 |
| **Medium Tree** | 0.70122 ± 0.072039 | 0.52727 ± 0.077663 | 0.62365 ± 0.060795 | 0.62838 ± 0.07142 | 0.62838 ± 0.07142 |
| **Coarse Tree** | 0.68537 ± 0.074191 | 0.57727 ± 0.08076 | 0.63716 ± 0.050234 | 0.6349 ± 0.052172 | 0.63483 ± 0.052223 |
| **Linear Discriminant Analysis** | 0.71951 ± 0.047808 | 0.58333 ± 0.056371 | 0.65878 ± 0.040392 | 0.65142 ± 0.040833 | 0.65142 ± 0.040833 |
| **Quadratic Discriminant Analysis** | 0.71463 ± 0.035477 | 0.58788 ± 0.064769 | 0.65811 ± 0.028108 | 0.65126 ± 0.030459 | 0.65126 ± 0.030459 |
| **Linear SVM** | 0.87561 ± 0.035301 | 0.34697 ± 0.065567 | 0.63986 ± 0.039282 | 0.74812 ± 0.027487 | 0.74812 ± 0.027487 |
| **Quadratic SVM** | 0.76829 ± 0.044413 | 0.55303 ± 0.062083 | 0.6723 ± 0.033065 | 0.74254 ± 0.027676 | 0.74254 ± 0.027676 |
| **Cubic SVM** | 0.74146 ± 0.030029 | 0.65606 ± 0.06688 | 0.70338 ± 0.030209 | 0.74435 ± 0.019104 | 0.74435 ± 0.019104 |
| **Fine Gaussian SVM** | 0.99634 ± 0.0089353 | 0.16061 ± 0.014247 | 0.62365 ± 0.006613 | 0.69946 ± 0.044738 | 0.69946 ± 0.044738 |
| **Medium Gaussian SVM** | 0.75488 ± 0.034013 | 0.8 ± 0.068399 | 0.775 ± 0.03879 | 0.81497 ± 0.023484 | 0.81497 ± 0.023484 |
| **Coarse Gaussian SVM** | 1 ± 0 | 0.0015152 ± 0.006776 | 0.55473 ± 0.0030217 | 0.73596 ± 0.03778 | 0.73596 ± 0.03778 |
| **Fine KNN** | 0.79878 ± 0.033456 | 0.50303 ± 0.034621 | 0.66689 ± 0.022898 | 0.65091 ± 0.022828 | 0.65091 ± 0.022828 |
| **Medium KNN** | 0.65732 ± 0.02795 | 0.71364 ± 0.046609 | 0.68243 ± 0.02214 | 0.70017 ± 0.01741 | 0.70017 ± 0.01741 |
| **Coarse KNN** | 1 ± 0 | 0 ± 0 | 0.55405 ± 1.1391e-16 | 0.4745 ± 0 | 0.47228 ± 1.1391e-16 |
| **Cosine KNN** | 0.88049 ± 0.035301 | 0.4 ± 0.048763 | 0.66622 ± 0.028784 | 0.76445 ± 0.027073 | 0.76445 ± 0.027073 |
| **Cubic KNN** | 0.64878 ± 0.022935 | 0.71667 ± 0.060025 | 0.67905 ± 0.030018 | 0.69834 ± 0.02761 | 0.69834 ± 0.02761 |
| **Weighted KNN** | 0.71829 ± 0.042962 | 0.66212 ± 0.050396 | 0.69324 ± 0.039484 | 0.74876 ± 0.022372 | 0.74876 ± 0.022372 |
| **Boosted Trees Emsemble** | 1 ± 0 | 0 ± 0 | 0.55405 ± 1.1391e-16 | 1 ± 0 | 0 ± 0 |
| **Bagged Trees Emsemble** | 0.78659 ± 0.066619 | 0.54394 ± 0.076456 | 0.67838 ± 0.039847 | 0.77683 ± 0.039493 | 0.77683 ± 0.039493 |
| **RUSBoosted Trees Ensemble** | 0.70976 ± 0.088083 | 0.52576 ± 0.10371 | 0.6277 ± 0.059387 | 0.6158 ± 0.050561 | 0.63452 ± 0.064263 |
| **Subspace Discriminant Ensemble** | 0.85488 ± 0.036671 | 0.27879 ± 0.063412 | 0.59797 ± 0.031578 | 0.72428 ± 0.032175 | 0.72428 ± 0.032175 |
| **Subspace KNN Ensemble** | 0.87317 ± 0.038442 | 0.43939 ± 0.077726 | 0.67973 ± 0.037741 | 0.83886 ± 0.031671 | 0.83886 ± 0.031671 |

**Table 3S:** Classification performance metrics for all models using SNV spectra to classify the control and occasional smokers’ group versus the smoker group.

|  | **Classification performance metric** | | | | |
| --- | --- | --- | --- | --- | --- |
| **Classification model** | **Specificity** | **Sensitivity** | **Accuracy** | **AUROC_Control & Occasional smokers** | **AUROCSmokers** |
| **Fine Tree** | 0.78235 ± 0.037447 | 0.59242 ± 0.10097 | 0.7203 ± 0.033518 | 0.71867 ± 0.051528 | 0.71861 ± 0.05161 |
| **Medium Tree** | 0.78382 ± 0.040231 | 0.59091 ± 0.069867 | 0.72079 ± 0.034087 | 0.70253 ± 0.046573 | 0.70256 ± 0.046599 |
| **Coarse Tree** | 0.79118 ± 0.046894 | 0.57121 ± 0.09546 | 0.71931 ± 0.040921 | 0.72306 ± 0.036343 | 0.72306 ± 0.036343 |
| **Linear Discriminant Analysis** | 0.73162 ± 0.048628 | 0.6 ± 0.075262 | 0.68861 ± 0.036973 | 0.66581 ± 0.040687 | 0.66581 ± 0.040687 |
| **Quadratic Discriminant Analysis** | 0.74779 ± 0.040842 | 0.61212 ± 0.06854 | 0.70347 ± 0.034817 | 0.67996 ± 0.039241 | 0.67996 ± 0.039241 |
| **Linear SVM** | 0.87426 ± 0.028816 | 0.59394 ± 0.071845 | 0.78267 ± 0.032359 | 0.85047 ± 0.019892 | 0.85047 ± 0.019892 |
| **Quadratic SVM** | 0.88162 ± 0.02546 | 0.67121 ± 0.04427 | 0.81287 ± 0.021284 | 0.8539 ± 0.025259 | 0.8539 ± 0.025259 |
| **Cubic SVM** | 0.82794 ± 0.032745 | 0.60152 ± 0.055854 | 0.75396 ± 0.028238 | 0.78819 ± 0.037904 | 0.78819 ± 0.037904 |
| **Fine Gaussian SVM** | 0.94044 ± 0.025903 | 0.19545 ± 0.045561 | 0.69703 ± 0.025375 | 0.74559 ± 0.025612 | 0.74559 ± 0.025612 |
| **Medium Gaussian SVM** | 0.95515 ± 0.028418 | 0.20758 ± 0.060025 | 0.71089 ± 0.026606 | 0.80711 ± 0.030174 | 0.80711 ± 0.030174 |
| **Coarse Gaussian SVM** | 1 ± 0 | 0 ± 0 | 0.67327 ± 1.1391e-16 | 0.78333 ± 0.019012 | 0.78333 ± 0.019012 |
| **Fine KNN** | 0.76471 ± 0.025247 | 0.47879 ± 0.061869 | 0.67129 ± 0.030237 | 0.62175 ± 0.036913 | 0.62175 ± 0.036913 |
| **Medium KNN** | 0.89485 ± 0.020397 | 0.30606 ± 0.066609 | 0.70248 ± 0.027535 | 0.75272 ± 0.024736 | 0.75272 ± 0.024736 |
| **Coarse KNN** | 1 ± 0 | 0 ± 0 | 0.67327 ± 1.1391e-16 | 0.47906 ± 2.8477e-16 | 0.47906 ± 2.8477e-16 |
| **Cosine KNN** | 0.80882 ± 0.036022 | 0.50455 ± 0.083583 | 0.70941 ± 0.031024 | 0.7495 ± 0.023954 | 0.7495 ± 0.023954 |
| **Cubic KNN** | 0.87279 ± 0.030653 | 0.30606 ± 0.062876 | 0.68762 ± 0.031877 | 0.73262 ± 0.027912 | 0.73262 ± 0.027912 |
| **Weighted KNN** | 0.88088 ± 0.025202 | 0.37727 ± 0.055158 | 0.71634 ± 0.024522 | 0.7714 ± 0.024594 | 0.7714 ± 0.024594 |
| **Boosted Trees Emsemble** | 1 ± 0 | 0 ± 0 | 0.67327 ± 1.1391e-16 | 1 ± 0 | 0 ± 0 |
| **Bagged Trees Emsemble** | 0.82059 ± 0.031864 | 0.53333 ± 0.050516 | 0.72673 ± 0.022348 | 0.78623 ± 0.025151 | 0.78623 ± 0.025151 |
| **RUSBoosted Trees Ensemble** | 0.76103 ± 0.037226 | 0.67576 ± 0.054828 | 0.73317 ± 0.028092 | 0.78007 ± 0.035053 | 0.78866 ± 0.026594 |
| **Subspace Discriminant Ensemble** | 0.91103 ± 0.015442 | 0.55909 ± 0.047635 | 0.79604 ± 0.018285 | 0.85597 ± 0.013173 | 0.85597 ± 0.013173 |
| **Subspace KNN Ensemble** | 0.83456 ± 0.030504 | 0.4 ± 0.05349 | 0.69257 ± 0.029173 | 0.7112 ± 0.031758 | 0.7112 ± 0.031758 |

**Table 4S:** Classification performance metrics for all models using the second derivative of SNV spectra to classify the control and occasional smokers’ group versus the smoker group.

|  | **Classification performance metric** | | | | |
| --- | --- | --- | --- | --- | --- |
| **Classification model** | **Specificity** | **Sensitivity** | **Accuracy** | **AUROC_Control & Occasional smokers** | **AUROCSmokers** |
| **Fine Tree** | 0.79265 ± 0.047449 | 0.50152 ± 0.069154 | 0.69752 ± 0.037112 | 0.67814 ± 0.044502 | 0.67807 ± 0.044547 |
| **Medium Tree** | 0.80662 ± 0.054666 | 0.48333 ± 0.10656 | 0.70099 ± 0.046172 | 0.67211 ± 0.059893 | 0.67209 ± 0.059903 |
| **Coarse Tree** | 0.81397 ± 0.044317 | 0.51515 ± 0.084001 | 0.71634 ± 0.043369 | 0.66914 ± 0.05479 | 0.66914 ± 0.05479 |
| **Linear Discriminant Analysis** | 0.76618 ± 0.03404 | 0.47424 ± 0.063164 | 0.67079 ± 0.031289 | 0.62021 ± 0.036315 | 0.62021 ± 0.036315 |
| **Quadratic Discriminant Analysis** | 0.74706 ± 0.031504 | 0.46061 ± 0.060286 | 0.65347 ± 0.02294 | 0.60383 ± 0.02833 | 0.60383 ± 0.02833 |
| **Linear SVM** | 0.99044 ± 0.009865 | 0.10152 ± 0.043164 | 0.7 ± 0.017329 | 0.75599 ± 0.029534 | 0.75599 ± 0.029534 |
| **Quadratic SVM** | 0.96765 ± 0.013992 | 0.14545 ± 0.046739 | 0.69901 ± 0.020163 | 0.80541 ± 0.032108 | 0.80541 ± 0.032108 |
| **Cubic SVM** | 0.94265 ± 0.027367 | 0.16515 ± 0.048639 | 0.68861 ± 0.020437 | 0.77845 ± 0.031529 | 0.77845 ± 0.031529 |
| **Fine Gaussian SVM** | 0.97794 ± 0.0089261 | 0.12879 ± 0.02759 | 0.7005 ± 0.0090145 | 0.72594 ± 0.026159 | 0.72594 ± 0.026159 |
| **Medium Gaussian SVM** | 0.94559 ± 0.014393 | 0.20455 ± 0.03388 | 0.70347 ± 0.015564 | 0.79853 ± 0.011877 | 0.79853 ± 0.011877 |
| **Coarse Gaussian SVM** | 1 ± 0 | 0 ± 0 | 0.67327 ± 1.1391e-16 | 0.78382 ± 0.027505 | 0.78382 ± 0.027505 |
| **Fine KNN** | 0.82721 ± 0.020172 | 0.33939 ± 0.040057 | 0.66782 ± 0.018022 | 0.5833 ± 0.021626 | 0.5833 ± 0.021626 |
| **Medium KNN** | 0.83824 ± 0.037569 | 0.3803 ± 0.044487 | 0.68861 ± 0.030386 | 0.7299 ± 0.022804 | 0.7299 ± 0.022804 |
| **Coarse KNN** | 1 ± 0 | 0 ± 0 | 0.67327 ± 1.1391e-16 | 0.47906 ± 2.8477e-16 | 0.47906 ± 2.8477e-16 |
| **Cosine KNN** | 0.92721 ± 0.016162 | 0.22576 ± 0.042259 | 0.69802 ± 0.019671 | 0.78961 ± 0.018231 | 0.78961 ± 0.018231 |
| **Cubic KNN** | 0.85368 ± 0.028014 | 0.34545 ± 0.060924 | 0.68762 ± 0.026191 | 0.75273 ± 0.013351 | 0.75273 ± 0.013351 |
| **Weighted KNN** | 0.83456 ± 0.02427 | 0.35909 ± 0.082418 | 0.67921 ± 0.02391 | 0.73012 ± 0.016456 | 0.73012 ± 0.016456 |
| **Boosted Trees Emsemble** | 1 ± 0 | 0 ± 0 | 0.67327 ± 1.1391e-16 | 1 ± 0 | 0 ± 0 |
| **Bagged Trees Emsemble** | 0.90221 ± 0.036109 | 0.35455 ± 0.06303 | 0.72327 ± 0.034369 | 0.79212 ± 0.037128 | 0.79212 ± 0.037128 |
| **RUSBoosted Trees Ensemble** | 0.75368 ± 0.035021 | 0.63939 ± 0.066609 | 0.71634 ± 0.029664 | 0.75402 ± 0.027165 | 0.75617 ± 0.026621 |
| **Subspace Discriminant Ensemble** | 0.99779 ± 0.0053875 | 0.018182 ± 0.018129 | 0.67772 ± 0.0059882 | 0.77353 ± 0.031944 | 0.77353 ± 0.031944 |
| **Subspace KNN Ensemble** | 0.94779 ± 0.015442 | 0.15909 ± 0.050011 | 0.6901 ± 0.022509 | 0.75309 ± 0.036683 | 0.75309 ± 0.036683 |
